# Metabolic radiogenomics in lung cancer: associations between FDG PET image features and oncogenic signaling pathway alterations

**DOI:** 10.1038/s41598-020-70168-x

**Published:** 2020-08-06

**Authors:** Gahyun Kim, Jinho Kim, Hongui Cha, Woong-Yang Park, Jin Seok Ahn, Myung-Ju Ahn, Keunchil Park, Yong-Jin Park, Joon Young Choi, Kyung-Han Lee, Se-Hoon Lee, Seung Hwan Moon

**Affiliations:** 1 Samsung Genome Institute, Samsung Medical Center, Seoul, Republic of Korea; 2 Department of Health Sciences and Technology, SAIHST, Sungkyunkwan University, Seoul, Republic of Korea; 3 Samsung Genome Institute, Samsung Medical Center, Samsung Advanced Institute of Health Science and Technology, Department of Molecular Cell Biology, Sungkyunkwan University School of Medicine, Seoul, Republic of Korea; 4 Division of Hematology/Oncology, Department of Medicine, Samsung Medical Center, Sungkyunkwan University School of Medicine, Seoul, Republic of Korea; 5 Department of Nuclear Medicine and Molecular Imaging, Samsung Medical Center, Seoul, Republic of Korea

**Keywords:** Biomarkers, Medical research, Oncology

## Abstract

This study investigated the associations between image features extracted from tumor ^18^F-fluorodeoxyglucose (FDG) uptake and genetic alterations in patients with lung cancer. A total of 137 patients (age, 62.7 ± 10.2 years) who underwent FDG positron emission tomography/computed tomography (PET/CT) and targeted deep sequencing analysis for a tumor lesion, comprising 61 adenocarcinoma (ADC), 31 squamous cell carcinoma (SQCC), and 45 small cell lung cancer (SCLC) patients, were enrolled in this study. From the tumor lesions, 86 image features were extracted, and 381 genes were assessed. PET features were associated with genetic mutations: 41 genes with 24 features in ADC; 35 genes with 22 features in SQCC; and 43 genes with 25 features in SCLC (FDR < 0.05). Clusters based on PET features showed an association with alterations in oncogenic signaling pathways: Cell cycle and WNT signaling pathways in ADC (p = 0.023, p = 0.035, respectively); Cell cycle, p53, and WNT in SQCC (p = 0.045, 0.009, and 0.029, respectively); and TGFβ in SCLC (p = 0.030). In addition, SUV_peak_ and SUV_max_ were associated with a mutation of the TGFβ signaling pathway in ADC (FDR = 0.001, < 0.001). In this study, PET image features had significant associations with alterations in genes and oncogenic signaling pathways in patients with lung cancer.

## Introduction

Radiogenomics, merging medical imaging data and genomic information, has great potential in the era of personalized medicine^1–4^. Genomic information could enable physicians to select appropriate management strategies according to the genetic alterations in an individual patient with cancer^5^. Nevertheless, the application of genomic medicine in the field of oncology has shortcomings because tumors have genetic and phenotypic diversities even within a single mass^5–7^. Such intra-tumor heterogeneity eventually drives treatment failure and disease progression^7–9^. To overcome it, multiple and sequential biopsies should be conducted to identify all the genetic alterations within the whole tumor throughout the course of disease progression within a patient, which is not always feasible in routine clinical practice. Therefore, the search for noninvasive techniques that reflect genetic alterations in multiple sites and at multiple time points is important because such techniques could improve patient care. Various features from medical images of a tumor could be surrogate markers that predict the alteration of particular genetic pathways. A known association between image features and genetic alterations has a potential as a useful additional information to improve decision making of biopsies, which could create new, accessible management strategies for patients with cancer^4,6,10^.

Associations between image features extracted from ^18^F-fluorodeoxyglucose (FDG) positron emission tomography/computed tomography (PET/CT) and genetic alterations have not been fully investigated. Image features derived from tumor FDG uptake, which reflects a tumor’s metabolic status, might be associated with the tumor’s genetic characteristics, and such an association could complement the radiogenomic approach made possible by anatomical imaging modalities. We previously investigated the associations among genetic characteristics, heterogeneity, mutation burden, and FDG PET/CT features in patients with lung cancer^11^. However, that study did not consider whether FDG PET indices are associated with particular genetic alterations. Despite the potential of radiogenomics-based FDG PET images, only a few studies have focused on the relationships between FDG uptake and genetic alterations in patients with lung cancer^12–15^, and the study subjects and PET image features of the few studies that have been done were not sufficient to fully elucidate the association between FDG PET imaging and genomic information^12–15^. Therefore, the value of features extracted from tumor FDG uptake in predicting genetic alterations in tumors has not been established. Whether or not PET-derived features can reflect alterations in specific oncogenic pathways is not well known.

Therefore, we investigated the associations between features extracted from tumor FDG uptake and genetic alterations in patients with lung cancer.

## Results

### Characteristics of subjects

The characteristics of the study patients are summarized in Table 1. They had a mean age (± standard deviation) of 62.7 ± 10.2 years (range, 29–82 years), and ADC was the most common cancer type (44.5%).

**Table 1:** Characteristics of Study Patients (n = 137).

Characteristics	n (%)
Age, years (range)	62.7 ± 10.2 (29–82)
Male	101 (73.7%)
**Smoking**	
Smoker	51 (37.2%)
Ex-smoker	46 (33.6%)
Never-smoker	39 (28.5%)
N/A	1 (00.7%)
**ECOG performance status**	
0	17 (12.4%)
1	102 (74.5%)
2	8 (05.8%)
3−4	1 (00.7%)
N/A	9 (06.6%)
**Pathology**	
ADC	61 (44.5%)
SQCC	31 (22.6%)
SCLC	45 (32.8%)
**AJCC TNM staging 7th**	
*T*	
1	19 (13.9%)
2	54 (39.4%)
3	34 (24.8%)
4	20 (14.6%)
N/A	10 (07.3%)
*N*	
0	36 (26.3%)
1	17 (12.4%)
2	38 (27.7%)
3	39 (28.5%)
N/A	7 (05.1%)
*M*	
0	75 (54.7%)
1a	12 (08.8%)
1b	27 (19.7%)
N/A	23 (16.8%)

ECOG, Eastern Cooperative Oncology Group; N/A, not applicable; ADC, adenocarcinoma; SQCC, squamous cell carcinoma; SCLC, small cell lung cancer; AJCC, American Joint Committee on Cancer; T, tumor; N, node; M, metastasis.

The 137 patients underwent 1st line surgery treatments only (29 patients, 21.2%), chemotherapy only (54 patients, 39.4%), chemoradiotherapy (16 patients, 11.7%), surgery combined with adjuvant chemotherapy (24 patients, 17.5%), surgery combined with chemoradiotherapy (10 patients, 7.3%), and surgery with neoadjuvant chemoradiotherapy (4, 2.9%).

Patients were clinically followed-up for a median of 24.5 months (range, 0.3–133.0 months). Of the 137 patients, 76 patients (55.5%) died during the follow-up period, and 105 patients (76.6%) had disease progression, with a median PFS of 8.7 months.

Among the 137 tumor lesions, 106 lesions were from lung tissue or bronchus (77.4%, 106/137), and 26 lesions were from lymph nodes (18.9%, 26/137). The remaining 5 tumor lesions were from pleura or trachea (4%, 5/137). In the 106 lung tissue or bronchus lesions, 64 were obtained by surgical resection (60.4%, 64/106), 29 were percutaneous needle biopsy (27.3%, 29/106), and the other 13 were obtained by bronchoscopy biopsy (12.3%, 13/106). All lymph node lesions were obtained by fine needle aspiration biopsy. On average, tumor lesions of enrolled patients had a SUV_max_ of 11.8 ± 5.7 and an MTV of 65.7 ± 89.2 cm^3^ (These are measured values before the correction by harmonization method).

### Genetic mutations and PET image features

We found that genetic mutations have an association with PET image features. Patients with mutations of particular genes showed different values in certain image features than patients without a mutation in those genes.

Due to limited space, the full dataset of the associations between PET image features and genetic mutations is presented only in Supplementary data 3 (FDR values). Briefly, in patients with ADC, 41 of the 381 target genes had an association with PET image features. The association varied from genes associated with 1 image feature to genes associated with 7 features. In terms of the PET image features, 24 features were associated with genetic mutation, and the number of genes associated with a particular feature ranged from 1 to 7. In SQCC, 35 genes had an association with image features (range from 1 to 10), and 22 image features had an association with genes (range from 1 to 9). In SCLC, 43 genes had an association with image features (range from 1 to 7), and 25 image features had an association with genes (range from 1 to 9).

The major associations (those genes which were mutated in more than 3 of the enrolled patients) are summarized in Table 2. Only 1 of the 41 associated genes in ADC, 4 of the 35 genes in SQCC, and 2 of the 43 genes in SCLC were mutated in more than 3 enrolled patients. In ADC, the PTCH2 gene were associated with 1 PET image feature; in SQCC, the ERCC2, IRS2, NOTCH1, and XPO1 genes were associated with 6 features; and in SCLC, the TSHR and ROS1 genes were associated with 2 features. Additionally, we calculated correlation of the PET image features in Supplementary data 4 because there are associations between the features.

**Table 2:** Correlation between gene mutations* and metabolic image features^†^.

Tumor	Mutation (number of related features)	Image features (FDR value)					
		GLCM normalized contrast					
ADC	PTCH2 (1)	0.029					

		SUV_max_	SUL_peak_	Surface SUV SD	Entropy	GLRM short run emphasis	TFCCM inverse difference moment
SQCC	ERCC2 (1)		0.037				
	IRS2 (2)	0.002		0.049			
	NOTCH1 (2)					0.029	0.033
	XPO1 (1)				0.001		

		NGLD small number emphasis	TFC mean convergence				
SCLC	TSHR (1)		0.036				
	ROS1 (1)	0.048					

*, Consisted only of genes having mutation over 3 cases; ^†^, consisted only of features showing significant correlation in FDR; ADC, adenocarcinoma; LN, lymph node; SQCC, squamous cell carcinoma; SCLC, small cell lung cancer; GLCM, gray level co-occurrence matrix; SUV, standardized uptake value; GLRM, gray level run-length matrix; TFCCM, texture feature coding co-occurrence matrix; NGLD, neighboring gray level dependence; TFC, texture feature coding.

### Clusters of image features and oncogenic signaling pathways

**Figure 1:**
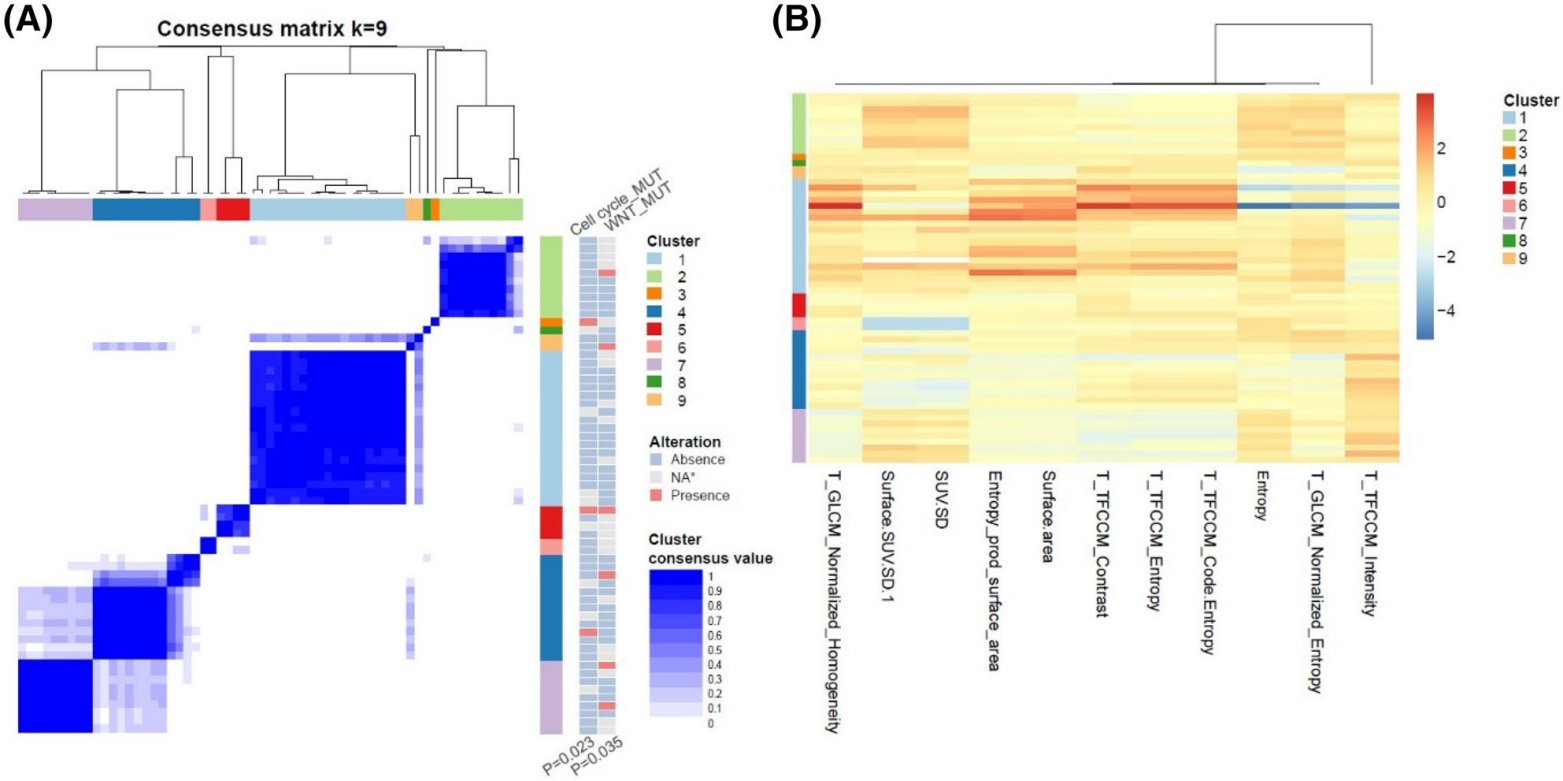
Visualization of the association between pathway alterations and clusters based on FDG PET features in ADC. (**A**) Patient clustering according to PET image features. The pathways on the right shows different incidence of alterations according to the clusters. (**B**) Heatmaps showing z-score of representative PET image features that were chosen based on statistical difference between clusters. MUT, mutation; NA, non applicable; T, texture feature; GLCM, gray level co-occurrence matrix; SUV, standardized uptake value; TFCCM, texture feature coding co-occurrence matrix.

**Figure 2:**
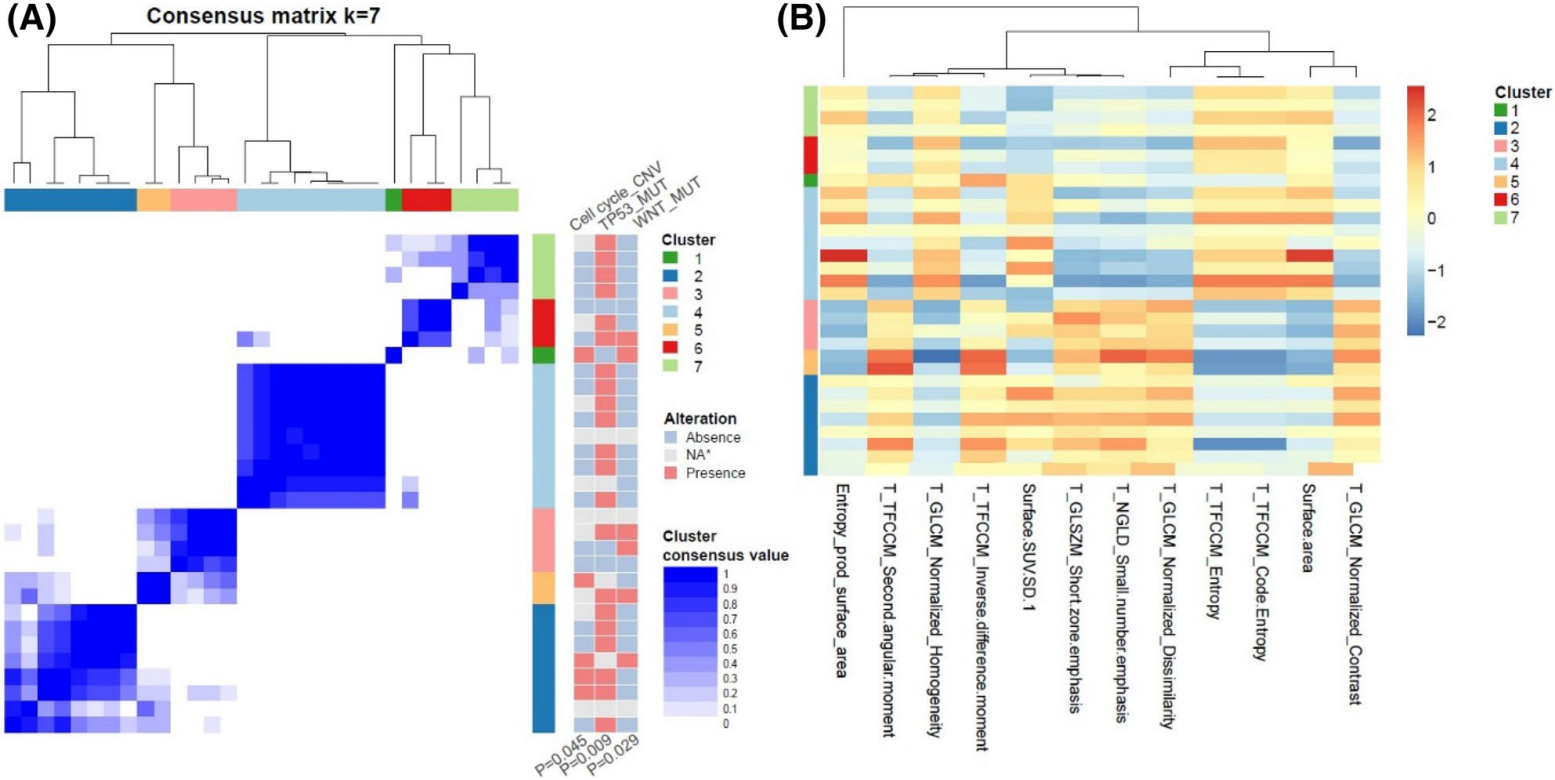
Visualization of the association between pathway alterations and clusters based on FDG PET features in SQCC. (**A**) Patient clustering according to PET image features. The pathways on the right shows different incidence of alterations according to the clusters. (**B**) Heatmaps showing z-score of representative PET image features that were chosen based on statistical difference between clusters. CNV, copy number variation, MUT, mutation; NA, non applicable; T, texture feature; TFCCM, texture feature coding co-occurrence matrix; GLCM, gray level co-occurrence matrix; SUV, standardized uptake value; SD, standard deviation; GLSZM, gray level size zone matrix; NGLD, neighboring gray level dependence.

**Figure 3:**
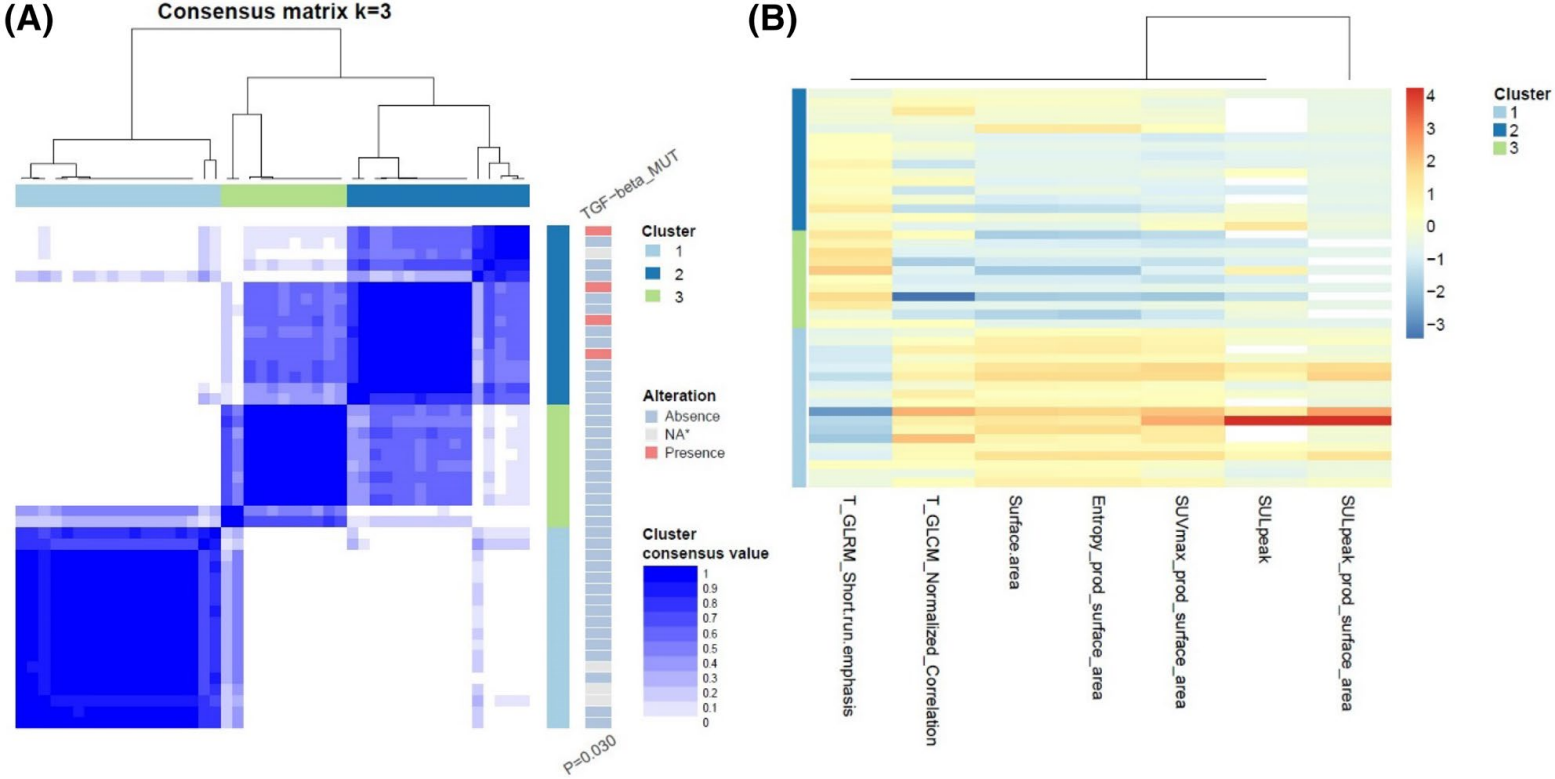
Visualization of the association between pathway alterations and clusters based on FDG PET features in SCLC. (**A**) Patient clustering according to PET image features. The pathways on the right shows different incidence of alterations according to the clusters. (**B**) Heatmaps showing z-score of representative PET image features that were chosen based on statistical difference between clusters. MUT, mutation; NA, non applicable; T, texture feature; T, texture feature; GLCM, gray level co-occurrence matrix; SUV, standardized uptake value; SUL; SUV normalized to lean body mass.

The clusters based on PET image features showed an association with alterations in the oncogenic signaling pathways. We classified patients into different groups on the basis of patterns of PET image features by using a consensus clustering analysis, and then we assessed the association between those groups and oncogenic signaling pathways. The results are presented as heatmaps in Figs. 1, 2, and 3, which supports that PET radiomic signature has a potential to predict specific oncogenic pathway alteration. Although the current form of the result has a limitation in practical aspect, they can be based to build a novel image-based model for predicting genetic alteration. In ADC, patients were divided into 9 different clusters, which were mainly represented by 11 PET image features, and the incidence of mutations in the cell cycle and WNT signaling pathways differed significantly by cluster (p = 0.023, p = 0.035, respectively, Fig. 1). The patients with SQCC were divided into 7 clusters represented by 12 features. Those clusters had associations with the cell cycle, p53, and WNT pathway (p = 0.045, 0.009, and 0.029, respectively, Fig. 2). The patients with SCLC divided into 3 clusters represented by 7 features, which were associated with the TGFβ signaling pathways (p = 0.030, Fig. 3).

**Figure 4:**
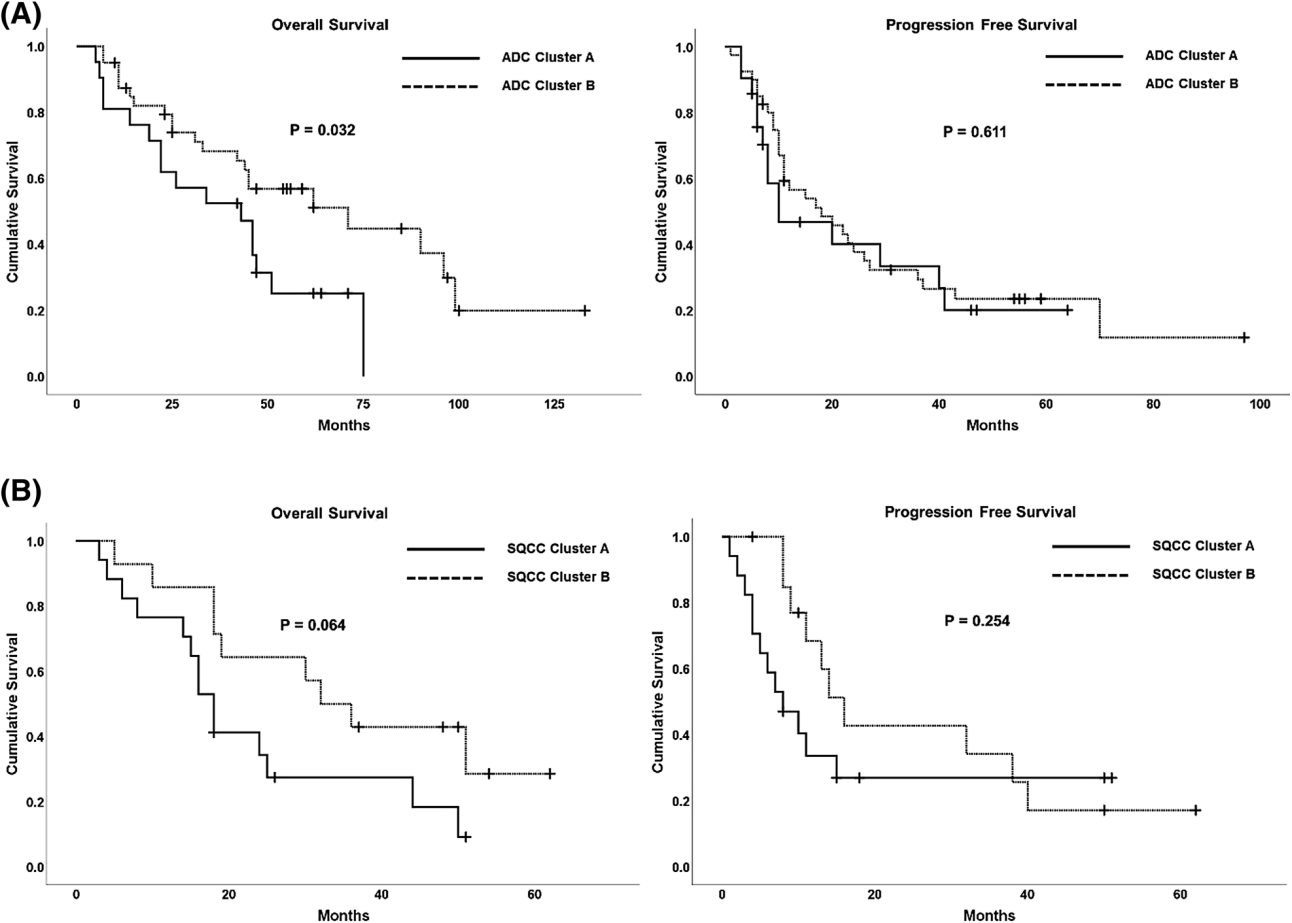
Prognostic value of the clusters in lung cancer. (**A**) OS differed significantly according to the reclassified clusters in ADC. The cluster A consists of the 1 and 9. The cluster B consists of the others (refer to Fig. 1). (**B**) In SQCC, there was no significant difference in survival according to the reclassified clusters. The cluster A consists of the 1, 4, 6, and 7. The cluster B consists of the 2, 3, and 5 (refer to Fig. 2). In SCLC, the reclassified cluster A of SCLC is the initial cluster 1 and the cluster B consists of the 2 and 3 (refer to Fig. 3), and there was also no significant difference in OS and PFS according to the reclassified clusters (Graph not shown, P = 0.194, P = 0.668, respectively). ADC, adenocarcinoma; SQCC, squamous cell carcinoma; SCLC, small cell lung cancer; OS, overall survival, PFS; progression free survival.

In addition, we assessed the survival value of the clusters. We selected the typical clusters based on their proximity to one another and the pattern of pathway alteration, and reclassified the clusters. The estimated survival over time according to the reclassified clusters is presented in Fig. 4. A Kaplan–Meier analysis showed that the patients in clusters 1 and 9 had better OS than the patients in the other clusters (p = 0.032) in ADC. Patients with SQCC and SCLC didn’t show any significant difference in survival according to the reclassified clusters. In addition, to evaluate survival value of the clusters in ADC, in relation to other factors such as TNM staging, therapeutic regimen, and other PET features, we performed a multivariate survival analysis using Cox proportional hazards regression model. However, the clusters didn't show any significance in multivariate analysis (supplementary table 5).

### Metabolic intensity and oncogenic signaling pathways

Metabolic intensity is associated with a mutation of the TGFβ signaling pathway in ADC. SUV_peak_ was selected to indicate the metabolic intensity of tumor lesions and was found to be higher in patients with mutations in the TGFβ signaling pathway than in those without them. When SUV_max_ was used as an indicator of metabolic intensity, the results were similar to those with SUV_peak_ in the TGFβ signaling pathway (fold change = 0.130, FDR < 0.001, data not shown). SUV_peak_ also showed raw p-value less than 0.05 in correlation with PI3-Kinase mutation. However, they failed to show significance in the FDR method.

In SQCC, although SUV_peak_ showed raw p-values less than 0.05 in correlation with mutation of the Hippo signaling pathway, they lost significance when they were corrected by the FDR method. In SCLC, metabolic intensity was not associated with alterations in oncogenic signaling pathways. The correlations between metabolic intensity, presented as SUV_peak_, and alterations in oncogenic signaling pathways are summarized in Table 3.

**Table 3:** Correlations between metabolic intensity and oncogenic signaling pathways according to pathology.

Pathways	ADC			SQCC			SCLC		
	p value	FC	FDR	p value	FC	FDR	p value	FC	FDR
**Cell cycle**									
Mutation	0.465	− 0.067	0.789	0.351	0.198	0.829	0.401	− 0.015	0.681
LOF	0.927	− 0.035	0.998	0.478	− 0.083	0.875	0.389	0.036	0.681
Fusion									
CNV	0.382	− 0.059	0.789	0.911	0.012	0.964	0.631	− 0.057	0.773
**Hippo**									
Mutation	0.239	0.007	0.763	0.038*	0.193	0.616	0.303	− 0.075	0.681
LOF									
Fusion									
CNV									
**Myc**									
Mutation							0.295	− 0.012	0.681
LOF									
Fusion									
CNV	0.951	− 0.075	0.998				0.031	0.132	0.596
**Notch**									
Mutation	0.117	− 0.009	0.530	0.676	− 0.033	0.875	0.242	− 0.100	0.675
LOF	0.998	− 0.078	0.998	0.318	− 0.080	0.829	0.107	0.228	0.596
Fusion									
CNV				0.292	− 0.136	0.829	0.718	− 0.023	0.789
**Nrf2**									
Mutation	0.254	− 0.162	0.763	0.901	− 0.011	0.964	0.529	0.417	0.711
LOF									
Fusion									
CNV	0.504	− 0.061	0.789	0.899	− 0.012	0.964			
**PI3-Kinase**									
Mutation	0.034*	− 0.145	0.408	0.982	0.001	0.982	0.455	− 0.022	0.692
LOF				0.659	− 0.069	0.875	0.993	− 0.091	0.993
Fusion									
CNV	0.434	− 0.014	0.789	0.195	0.113	0.805	0.168	0.146	0.596
**RTK/RAS-Kinase**									
Mutation	0.329	− 0.137	0.789	0.083	0.170	0.724	0.180	0.252	0.596
LOF									
Fusion									
CNV							0.183	0.086	0.596
**TGFβ**									
Mutation	< 0.001	0.101	0.001*				0.479	− 0.158	0.692
LOF	0.118	− 0.161	0.529						
Fusion									
CNV							0.634	− 0.045	0.773
**p53**									
Mutation	0.072	0.119	0.529	0.113	0.135	0.724	0.473	− 0.069	0.692
LOF	0.469	− 0.027	0.789	0.281	− 0.106	0.829	0.142	0.199	0.596
Fusion									
CNV	0.848	− 0.048	0.998	0.694	− 0.056	0.875			
**β-catenin/Wnt**									
Mutation LOF Fusion CNV	0.953	− 0.059	0.998	0.581	− 0.067	0.875	0.709	0.198	0.789
	0.643	− 0.022	0.626						
							0.831	− 0.055	0.876

*Significant correlation; ADC, adenocarcinoma; SQCC, squamous cell carcinoma; SCLC, small cell lung cancer; FC, fold change; FDR, false discovery rate; LOF, loss of function; CNV, copy number variation.

## Discussion

We have here investigated the relationships between metabolic image features and genomic alterations, including oncogenic signaling pathways, from the dataset of our institution. Although the limitations of a heterogeneous dataset and incomplete methodology have hampered our ability to generalize our findings, some results are consistent with those of previous studies indicating that the FDG PET-based radiogenomic approach has the potential to play a significant role in cancer research and clinical practice.

In recent years, the search for relationships between imaging phenotypes and genomics, commonly called radiogenomics or image genomics, has emerged as a new direction in cancer research. Knowledge from this new approach has could lead to improved decision making in the management of cancer patients^4,10,11^. FDG PET-derived image features can reflect the metabolic status of a tumor in ways that anatomical imaging modalities cannot^16,17^. Genomic alterations underlie a pan-cancer metabolic shift, which is one of the hallmarks of cancer^18,19^. Many key neoplastic events are controlled by a host of mutational events in multiple cancer-associated genes that converge to alter tumor cell metabolism^19–21^. Therefore, there might be some relationships between the characteristics of tumor FDG uptake and the alteration of cancer-associated genes and pathways. However, despite the research interest in radiogenomics, the value of using an image analysis based on tumor FDG uptake to predict genetic alterations has not been established. Particularly, very few studies have focused on FDG PET–based radiogenomics in lung cancer^12–14^. Nair VS et al. explored differential genome-wide expression across 14 different SUV histogram features in 25 patients with non-small cell lung cancer (NSCLC) to identify individual genes and gene expression signatures associated with prognostically relevant features^12^. They reported that 8 single genes (BIRC2, FAP, FURIN, LOC648470, LY6E, MCM6, RNF149, and OBFC1) were associated with 7 different SUV histogram features, and eight co-expressed gene clusters (cell adhesion, protein catabolism, nucleic acid processing, metalloproteinase, TP53, RB1, protein processing, embryogenesis, apoptosis, extracellular matrix, and hypoxia ) were also associated with 7 different features. Gevaert O et al. also applied a radiogenomic approach to a cohort of 26 patients with NSCLC^13^. They extracted 180 image features from CT (179 features) and PET/CT (SUV_max_), and identified 243 significant pairwise correlations between image features and co-expressed gene clusters in NSCLC. Among them, SUV_max_ was associated with 4 co-expressed gene clusters. Crespo-Jara A et al. developed a universal genomic signature predicting the FDG uptake of 84 patients with diverse metastatic tumors, including 7 with lung cancer^14^. They found that SUV_mean_ correlated with biological processes beyond glycolysis, ribosome biogenesis, DNA replication, cytoskeleton reorganization, and autophagy. Although those researchers have elucidated meaningful results from the data available at the time in a sophisticated way, the relevance of PET image features to predicting gene alteration has not been proved. The patient populations included in the previous studies were too small to have sufficient statistical power. In addition, only a few of the many PET image features were considered^12–14^.

We explored differential genes and oncogenic signaling alterations across 27 PET features (SUV histogram features and textures features) in a cohort of 137 patients with lung cancer (61 ADC, 31 SQCC, and 45 SCLC patients) to identify individual genes and oncogenic signaling pathways associated with PET image features. One strength of the present study is that it considers a much larger study population than previous studies. Another is that it includes a much larger number of PET image features in its analysis. On the other hand, the number of genes analyzed in this study is relatively small compared with previous studies. We used data from a cohort of patients who underwent CancerSCAN, which targeted 83 or 381 genes, which is too few to identify hidden molecular mechanisms or pathways associated with tumor metabolism. In addition, mutation in a single gene may not be enough to guarantee an alteration in the specific pathway it belongs to, which may have led to misinterpretation in the present study. However, the selected target genes are associated with targeted cancer therapies or response to therapy in the literature and public databases^22,23^, Our data are, therefore, sufficient to identify clinically meaningful associations between genetic alterations and image features.

In this study, 41 single genes were associated with 24 PET image features in ADC. Among them, the only one gene, PTCH2, was arbitrarily classified according to the frequency of alteration among the study subjects. It has known to act as a tumor suppressor^24^, and showed significant association with GLCM normalized contrast in this study. In addition, image clusters comprising 11 features were associated with the alteration of the cell cycle and WNT signaling pathway. The association of PET features with single genes reported in a previous study was not reproduced in this study^12^. However, it is difficult to make a direct comparison with the result of the previous study because various factors that can affect the result, such as the PET image features used, the applied target genes, tumor segmentation method, scanner, image protocol, and analyzing strategy are all different. In SQCC, four major genes were associated with 6 PET image features: ERCC2^25^, IRS2^26^, NOTCH1^27^, and XPO1^28^, which functions as a DNA repair gene^25^, mediates effects of insulin and other cytokines^26^, plays a role in cell growth, division, differentiation, and apoptosis^27^, and mediates the nuclear export of proteins and RNAs^28^, respectively. Image clusters were associated with the p53, WNT, and cell cycle pathways. In SCLC, two major genes were associated with 2 PET image features: TSHR^29^ and ROS1^30^, which mediates thyroid cell metabolism^29^ and functions as a growth or differentiation factor receptor^30^, respectively. Image clusters were associated with the TGFβ signaling pathways. To the best of our knowledge, this is the first study to assess associations between genetic alterations and PET image features, including texture features, in SQCC and SCLC.

However, it is quite questionable whether the association between the image feature/clusters and gene mutation will be reproduced in another cohort of patients. Many factors create obstacles to generalizing the findings of this study, but one of the biggest problems is that the image features are susceptible to various conditions and thus are not a robust indicator that produces consistent outcomes under different study conditions. PET image features are significantly affected by tumor size^31^, tumor segmentation method^11,32^, scanner, and image protocol^33^. In addition, measurement of the PET texture features is 5 times more sensitive to volume changes for small volumes below the proposed minimum than for those above it^34^. The textural features also depend on the conditions of image acquisition and the reconstruction method. Although there was a difference in degree, all such features exhibited variations according to different acquisition modes and reconstruction parameters^17,35,36^. Standardization and refinement of methodologies for producing objective and independent image features is required to ensure results of radiomic studies^37^. Consequently, unless features are obtained with a similar tumor size and the same segmentation method, acquisition modes, and reconstruction parameters, studies such as this one are difficult to compare with other studies. However, a collaboration works toward for standardization such as the image biomarker standardisation initiative (IBSI) has been proposed. Efforts toward standardizing all of these technical issues for the radiomic approach has been made^6^, which will facilitate the comparison of results between these kinds of studies and will be the basis for drawing reliable conclusions. In this study, to minimize the effect of PET scanners and segmentation methods, we adopted ComBat for harmonization^38^ recommended by IBSI and choose the robust features for segmentation methods^11^.

Even though the measurement of PET features is generally sensitive to various conditions, some indicators are relatively robust and can be used with some degree of certainty. SUV_peak_ and SUV_max_, which indicate the metabolic intensity of the target tumor lesion, provide an observer-independent measurement and are less affected by conditions than other features^39,40^. These intensity features are related to certain genes or oncogenic signaling pathways and are likely to have potential as a universal surrogate marker that is reproducible even under different conditions. In this study, SUV_peak_ was associated with the TGFβ signaling pathway in ADC. This pathway is an important mediator of tumor invasion, and targeted inhibition of this pathway could be a new approach to lung cancer treatment^41–43^. This result is consistent with that of a previous study. Yamamoto S et al. conducted a differential expression analysis in a public NSCLC dataset that contained FDG PET and messenger RNA expression profile data (n = 26). They found that the genes which have been strongly implicated in epithelial-mesenchymal transition (EMT), including TGF-β (P-value = 0.007) were overexpressed in their high-normalized SUV_max_ group^15^. The results from both studies demonstrate a strong association between increased FDG uptake and gene alterations related to EMT in ADC. Given that SUV_max_ is indicator of relatively low variability that are not significantly affected by other conditions, the conclusion in that TGF-β signaling alteration are more likely to occur in ADC patient with high metabolism is highly reliable. Of course, it should be further confirmed in larger size populations.

This study has several limitations. First, because of an insufficient number of enrolled study subjects, internal validation was not performed, which diminished the quality of the research. Second, many of extracted features using CGITA were not identical to those provided by IBSI, which limits the generalizability and reproducibility of this study. In addition, the heterogeneity of the study subjects, including the scanner, reconstruction method, version of CancerSCAN, and different tissue type could restrict the generalizability of our study results. Validation studies with a large number of homogeneous subjects and standardized radiomic PET features are needed to confirm the findings of this study.

In conclusion, we found significant associations between PET image features and gene mutations in this study. Interestingly, the metabolic intensities of the tumor, presented as SUV_peak_ and SUV_max_, and clusters of PET image features were associated with alterations in specific oncogenic signaling pathways, suggesting that it is not impossible to predict the presence of an alteration by using FDG PET in patients with lung cancer.

However, it is difficult to generalize our results due to the limitations given above and the methodological challenges of metabolic radiogenomics, including the reproducibility and reliability of the features. Further large-scale studies are needed to verify the findings of this study and evaluate whether PET image have predictive value for genetic alteration.

## Methods

### Subjects

**Figure 5:**
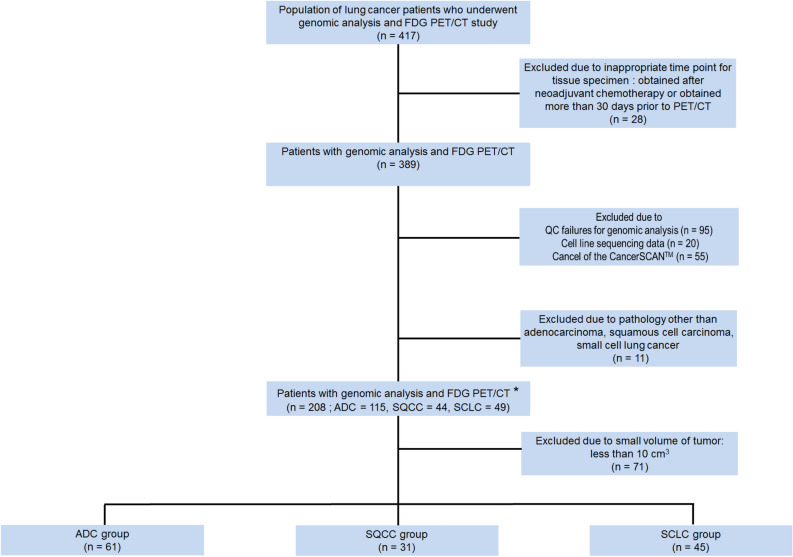
Flowchart of patient inclusion, with reasons for exclusion and the total study population. *, These patients were included in the analysis for the correlation between the intensity of tumor FDG uptake and alterations in oncogenic signaling pathways.

In this study, we enrolled novel study subjects from the candidates for our previous study^11^. The process of subject selection is summarized in Fig. 5. Briefly, the study candidates were 417 patients with histologically confirmed lung cancer who were enrolled in a database at the Samsung Genome Institute and who underwent ^18^F-FDG PET/CT. Gene profiles of their tumor tissue had supposed to be made using the CancerSCAN next generation sequencing (NGS)-based targeted-sequencing platform designed at our institution^44^. All patients had agreed that their data could be used in other studies. Within that candidate pool, we excluded 28 patients whose tumor tissues were obtained for genomic analysis after neoadjuvant therapy or more than 30 days prior to their PET/CT.

Of the remaining 389 patients, we excluded 95 patients whose CancerSCAN results failed quality control, 20 patients with cell line sequencing data, 55 patients who cancelled the CancerSCAN, and 11 patients with cancers other than adenocarcinoma (ADC), squamous cell carcinoma (SQCC), or small cell lung cancer (SCLC). All PET scans in these patients was performed before treatment. In addition, we excluded 75 patients with small tumor volume because small tumor volume affects the measurement of texture features in PET images^31^. The minimum metabolic tumor volume eligible for texture feature analysis is approximately 10 cm^3^^11,45^. Thus, patients with less than 10 cm^3^ of tumor volume were excluded from the analysis. Therefore, a total of 137 patients were finally included and divided into 3 groups by their cancer type (ADC, SQCC, and SCLC).

All the clinical data for the enrolled patients were collected by a review of electronic medical records. Overall survival (OS) was defined as the time from the first date of 1st line treatment until death from any cause, with censoring at the date of the final follow-up in surviving patients. Progression-free survival (PFS) was defined as the time from the first date of 1st line treatment to the date of disease progression, with censoring at the date of the final follow-up if the patient had not progressed. This study was approved by Institutional Review Board of Samsung Medical Center, and the requirement for written informed consent was waived. In addition, all methods were performed in accordance with relevant guidelines and regulations.

### PET/CT imaging

All patients were instructed to fast for at least 6 h, and blood glucose was < 200 mg/dl at the time of the FDG injection. PET/CT scans without intravenous or oral contrast were performed on a GE Healthcare (Milwaukee, WI, USA) Discovery LS (n = 41) or Discovery STe (n = 96) scanner. At 60 min after injecting 225–417 MBq of FDG, emission scans were acquired from the skull base to mid-thigh at 4 min per frame in 2D mode (Discovery LS) or 2.5 min per frame in 3D mode (Discovery STe). Whole-body spiral CT was performed with an 8-slice helical CT (140 keV, 40 to 120 mAs adjusted to body weight; section width = 5 mm) for the Discovery LS scanner and a 16-slice helical CT (140 keV, 30 to 170 mAs with AutomA mode; section width = 3.75 mm) for the STe scanner. Attenuation-corrected PET images (voxel size = 4.3 × 4.3 × 3.9 mm for Discovery LS, 3.9 × 3.9 × 3.3 mm for Discovery STe) were reconstructed using CT data and 2D (28 subsets, 2 iterations; Discovery LS) or 3D ordered-subset expectation maximization algorithms (20 subsets, 2 iterations; STe).

### PET image analysis

To investigate the association between a PET image feature and a gene mutation or alteration in an oncogenic signaling pathway, we extracted PET image features from the tumors for which a tissue biopsy was performed using CancerSCAN.

Image feature extraction was based on a previous study and used the gradient-based segmentation method (‘PET Edge’) in MIM version 6.4 software (MIM Software Inc., Cleveland, OH, USA)^11,46^. The target tumor was identified by an experienced nuclear medicine physician (S.H.M) who was unaware of all clinical information except the target tumor site. As the physician drags the cursor out from the center of the target tumor to a point near the edge of the lesion, six axes interactively extend out, and the length of an axis is restricted when a large gradient is detected along that axis. Then, the software automatically outlines a three-dimensional volume of interest on the tumor. After creating gradient-based segmentation of the target tumor lesion, we extracted PET image features using the Chang-Gung Image Texture Analysis toolbox (CGITA, https://code.google.com/p/cigita), an open-source software package implemented in MATLAB (version 2012a; MathWorks Inc., Natick, MA, USA)^47^. A total of 86 PET features available in CGITA were measured on each segment^11,47^: 55 texture features and 31 non-texture features (standardized uptake value (SUV) and intensity histogram, 25; texture spectrum, 2; geometry, 4; see Supplementary data 1). To minimize the effect of different PET scanners with different image reconstruction method (2D vs. 3D), we adopted harmonization method which is recommended by IBSI (https://github.com/Jfortin1/Com-BatHarmonization)38, and obtained corrected values of extracted features. Among those corrected 86 image features, 27 features which have shown to be less affected by segmentation methods^11^ (Supplementary data 1) were selected for further analysis. The metabolic tumor volume (MTV), obtained through the gradient-based segmentation method, was used as a volume indicator to determine whether or not the measured target tumor would be included in the subject enrollment process (Fig. 5).

### CancerSCAN and classification of oncogenic signaling pathway

CancerSCAN is an NGS-based targeted-sequencing platform designed at our institution. The reliability of this assay was proved by a robust analytic validation in previous studies, where the details of experimental procedures were described^22,23,44^.

CancerSCAN version 1 targeted 83 genes, and version 2 targeted 381 genes. The selected target genes for this customized platform were curated at the request of researchers and clinicians and associated in the literature and public databases with targeted cancer therapies or response to therapy. SNVs, small indels, CNVs, and gene fusions were detected using both existing and new algorithms. The genes contained in the two versions are listed in Supplementary data 2. The variant calls were classified into four categories to reflect mode and functional effect of mutations and then condensed at gene level. The four categories include (1) MUT: miss-sense mutation, (2) LoF: loss of function variant including frame-shift insertion/deletion and stop-gain mutation, (3) CNV: copy number variation, and (4) FUSION: known driver gene fusion event.

The genes in CancerSCAN were classified into ten canonical pathways according to the mechanism and pattern of alterations: cell cycle, Hippo, Myc, Notch, NF-E2 p45-related factor 2 (Nrf2), phosphoinositide-3-kinase–protein kinase (PI-3-Kinase)/Akt, receptor tyrosine kinases (RTK)-RAS, transforming growth factor-β (TGFβ) signaling, p53, and β-catenin/Wnt signaling, as proposed by F Sanchez-Vega et al.^48^. The genes corresponding to those pathways are listed in Supplementary data 2. In this study, patients with alterations in any of the genes corresponding to a particular pathway were considered to have alterations in that pathway.

### Statistical analysis

Statistical analysis was performed using SAS version 9.4 (SAS Institute, Cary, NC, USA) and R 3.5.3 (Vienna, Austria; https://www.R-project.org/).

The associations between PET image features and genetic mutations and pathway alterations were assessed. In comparing texture features between patients with gene alterations and those without them, a total of 137 patients with tumor volume ≥ 10 cm^3^ were enrolled. In the comparison of FDG uptake intensity between patients with a pathway alteration and those without one, a total of 208 patients who met all the criteria of this study except for tumor volume were included because PET image features that represent FDG uptake intensity, such as SUV_peak_ or SUV_max_, are not much affected by tumor volume^11,31,45^.

The PET image features were verified to determine whether they have a normal distribution. If the values were not normally distributed, they were log-transformed. T-testing was used when the values of the image features were normally distributed; otherwise, the Wilcoxon rank sum test was used. To assess how much a quantity changed, we calculated log fold changes of mean PET image feature between the patients having the alteration and the others. Before calculating the log fold change, if either numerator or denominator equals zero, rescaling was applied as follows:

$$\frac{\frac{Xi}{Max(X)}\times \left(N-1\right)+0.5}{N},$$

where X is a PET image feature, and N is the number of the subjects. To correct random events that falsely appear significant, false discovery rate (FDR) was calculated using Benjamini Hochberg procedure, a statistical approach for multiple comparisons.

We clustered the study subjects based on all PET image features using consensus clustering analysis in which we used hierarchical clustering algorithm and Pearson correlation as distance metric. Based on the proportion of ambiguous clustering (PAC) with the interval of consensus index ranging from 0.1 to 0.9, we chose the optimal number of clusters (K). To identify the key image features that are distinctive between clusters, statistical tests were used; Firstly, either ANOVA, Welch’s ANOVA or Kruskal Wallis test depending on the normality of the distribution and homogeneity of variance. Secondly, pair-wise t-tests were performed and image features that were significant in all the tests were chosen in ADC and SQCC. In SCLC, there were no image features significant in all the 6 tests, thus we chose image features significant in five tests instead.

We also assessed the survival value of the clusters based on PET image features. The obtained clusters were reclassified according to their proximity to one another, which was based on dendrogram. The clusters split off together from the higher branches of the dendrogram were reclassified. They were defined in Figure Legends (Fig. 4). Progression free survival (PFS) and overall survival (OS) according to the reclassified clusters were estimated using the Kaplan–Meier survival analysis method and compared using the log-rank test.

All tests were two-sided, and p-values less than 0.05 were considered statistically significant.

## Supplementary information

Supplementary data legends

Supplementary data 1

Supplementary data 2

Supplementary data 3

Supplementary data 4

Supplementary data 5
