# Supplementary material for: Metabolic radiogenomics in lung cancer: associations between FDG PET image features and oncogenic signaling pathway alterations
Source: Sci Rep. 2020 Aug 6;10:13231. doi: 10.1038/s41598-020-70168-x (PMC7411040; doi:10.1038/s41598-020-70168-x)
Supplement: Supplementary file 1 — Supplementary data legends [file 41598_2020_70168_MOESM1_ESM.docx]

**Supplementary Data**

**Metabolic Radiogenomics in Lung Cancer: Associations Between FDG PET Image Features and Oncogenic Signaling Pathway Alterations**

Gahyun Kim^1,2,*^, Jinho Kim^1,*^, Hongui Cha^1,2^, Woong-Yang Park^3^,, Jin Seok Ahn^4^, Myung-Ju Ahn^4^, Keunchil Park^4^, Yong-Jin Park^5^, Joon Young Choi^5^, Kyung-Han Lee^5^, Se-Hoon Lee^2,4,†^, Seung Hwan Moon^5,†^

Supplementary data 1. PET image features

Supplementary data 2. Target genes and pathways

Supplementary data 3. Associations between PET image features and presence or absence of genetic mutations in ADC (3-1), SQCC (3-2), and SCLC (3-3).

Supplementary data 4. Visualization of correlation matrix between 27 image features significantly associated with gene mutations in Table 2.

Supplementary data 5. Result of the survival analysis using the Cox proportional hazards model in ADC
