# Supplementary figures and images for: Metabolic radiogenomics in lung cancer: associations between FDG PET image features and oncogenic signaling pathway alterations
Source: Sci Rep. 2020 Aug 6;10:13231. doi: 10.1038/s41598-020-70168-x (PMC7411040; doi:10.1038/s41598-020-70168-x)

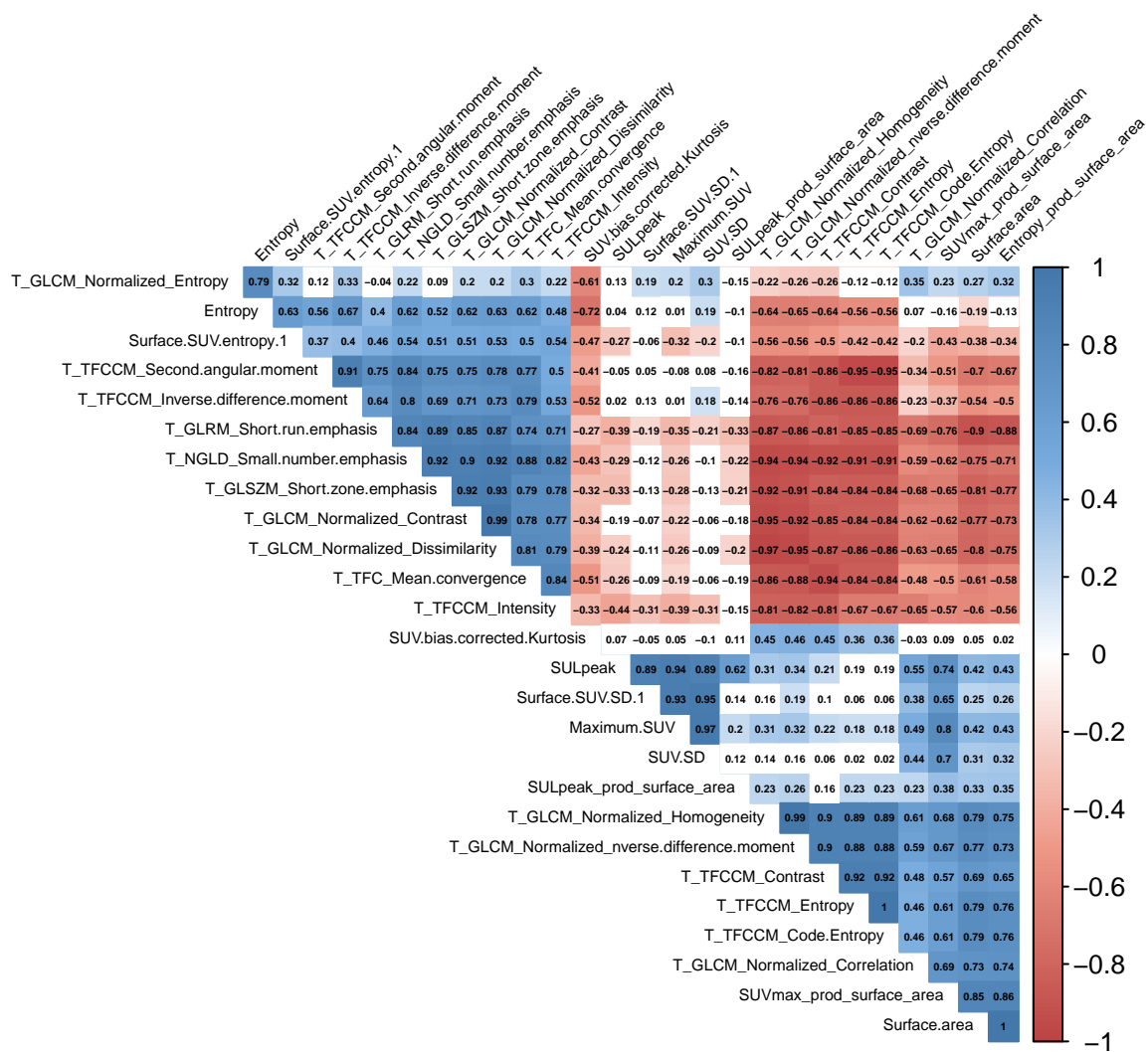

Supplement: Supplementary file 5 — Supplementary data 4 [file 41598_2020_70168_MOESM5_ESM.pdf]
