# Supplementary material for: Metabolic radiogenomics in lung cancer: associations between FDG PET image features and oncogenic signaling pathway alterations
Source: Sci Rep. 2020 Aug 6;10:13231. doi: 10.1038/s41598-020-70168-x (PMC7411040; doi:10.1038/s41598-020-70168-x)
Supplement: Supplementary file 6 — Supplementary data 5 [file 41598_2020_70168_MOESM6_ESM.docx]

Supplementary data 5. Result of the survival analysis using the Cox proportional hazards model in ADC

|  |  | Univariate | | |  | Multivariate | | |
| --- | --- | --- | --- | --- | --- | --- | --- | --- |
|  |  | OR | 95% CI | *P*-value |  | OR | 95% CI | *P*-value |
| Progression-free Survival | |  |  |  |  |  |  |  |
| Age ≥ 65 years (vs. < 65 years) | | 0.69 | 0.35-1.34 | 0.268 |  |  |  |  |
| Male (vs. female) | | 1.41 | 0.77-2.56 | 0.263 |  |  |  |  |
| Smoker (vs. never smoker) | | 2.08 | 1.01-4.30 | 0.048 |  |  |  |  |
| T stage | | 1.19 | 0.79-1.78 | 0.403 |  |  |  |  |
| N stage | | 1.23 | 0.95-1.60 | 0.115 |  |  |  |  |
| M stage | | 0.91 | 0.57-1.44 | 0.680 |  |  |  |  |
| CTx only (vs. surgery only) | | 1.24 | 0.46-3.34 | 0.672 |  |  |  |  |
| Cluster A (vs. cluster B) | | 1.18 | 0.62-2.23 | 0.618 |  |  |  |  |
| Overall Survival | |  |  |  |  |  |  |  |
| Age ≥ 65 years (vs. < 65 years) | | 1.95 | 0.97-3.90 | 0.059 |  |  |  |  |
| Male (vs. female) | | 1.31 | 0.68-2.51 | 0.417 |  |  |  |  |
| Smoker (vs. never smoker) | | 1.078 | 0.49-2.33 | 0.850 |  |  |  |  |
| T stage | | 1.24 | 0.83-1.87 | 0.293 |  |  |  |  |
| N stage | | 1.32 | 0.99-1.78 | 0.061 |  |  |  |  |
| M stage | | 1.81 | 1.21-2.72 | 0.004 |  |  |  |  |
| CTx only (vs. surgery only) | | 5.77 | 2.19-15.2 | <0.000 |  | 7.27 | 1.08-49.1 | 0.042 |
| Cluster A (vs. cluster B) | | 2.058 | 1.05-4.05 | 0.037 |  |  |  |  |
|  | |  |  |  |  |  |  |  |

ADC, adenocarcinoma; OR, odds ratio; CI, confidence interval; CTx, chemotherapy; CCRT, concurrent chemoradiation therapy
